# Impact of temporal resolution in single particle tracking analysis

**DOI:** 10.1186/s11671-024-04029-1

**Published:** 2024-05-09

**Authors:** Chiara Schirripa Spagnolo, Stefano Luin

**Affiliations:** 1 NEST Laboratory, Scuola Normale Superiore, Piazza San Silvestro 12, 56127 Pisa, Italy; 2 NEST Laboratory, Istituto Nanoscienze-CNR, Piazza San Silvestro 12, 56127 Pisa, Italy

**Keywords:** Single molecule tracking, Nanobioimaging, Molecular diffusion, Mean square displacement, Single molecule simulations

## Abstract

Temporal resolution is a key parameter in the observation of dynamic processes, as in the case of single molecules motions visualized in real time in two-dimensions by wide field (fluorescence) microscopy, but a systematic investigation of its effects in all the single particle tracking analysis steps is still lacking. Here we present tools to quantify its impact on the estimation of diffusivity and of its distribution using one of the most popular tracking software for biological applications on simulated data and movies. We found important shifts and different widths for diffusivity distributions, depending on the interplay of temporal sampling conditions with various parameters, such as simulated diffusivity, density of spots, signal-to-noise ratio, lengths of trajectories, and kind of boundaries in the simulation. We examined conditions starting from the ones of experiments on the fluorescently labelled receptor p75^NTR^, a relatively fast-diffusing membrane receptor (diffusivity around 0.5–1 µm^2^/s), visualized by TIRF microscopy on the basal membrane of living cells. From the analysis of the simulations, we identified the best conditions in cases similar to these ones; considering also the experiments, we could confirm a range of values of temporal resolution suitable for obtaining reliable diffusivity results. The procedure we present can be exploited in different single particle/molecule tracking applications to find an optimal temporal resolution.

**Supplementary Information:**

The online version contains supplementary material available at 10.1186/s11671-024-04029-1.

## Introduction

Single particle tracking (SPT) is a powerful technique that consists in the reconstruction of trajectories of single objects. It has been widely applied in life science in different biological contexts to extract valuable insight into the spatiotemporal behaviour of cells, organelles, molecules, and associated functions: from signalling, to transcription, to infection or drug delivery [1–6]. SPT combines high spatial and temporal resolution for a complete picture of the process under investigation.

SPT consists of two main steps: the first one includes particle detection and localization in space; the second one is the strictly “tracking” step with linking of the located spots through time [7–9]. Localization is performed at subpixel resolution, e.g. by Gaussian fitting of the spot intensity profile, obtaining typical accuracy of tens of nanometers, way below the standard resolution of the used microscope [10, 11]. Starting from this superresolved localization, information at the cellular or cellular compartment scale can be obtained, usually starting from the classification of the motion kind (e.g. confined, diffusive, drifted, subdiffusive) or simply by the measurement of the diffusion coefficient, typically extracted from the mean square displacement (MSD) function, eventually considering its distribution in different compartments or under different conditions [10, 12–15].

The effects of temporal resolution in SPT have mostly been investigated considering its influence on localization accuracy. Two kinds of uncertainties related to temporal resolution influence particle localization in SPT: static and dynamic [16–19]. The first one is the experimental-noise related uncertainty affecting the localization of particles, even if immobilized; the second one is caused by the motion of the particles during the acquisition time of each frame. Static errors increase for low signal-to-noise ratios (SNRs), dynamic errors (“blurring”) increase with diffusivity and integration time [20–22].

Instead, uncertainties strictly connected to the linking step have been much less investigated, and a complete analysis considering both localization and tracking effects on the accuracy of SPT analysis and diffusivity estimations under different conditions is still lacking. Here we perform a systematic investigation of the effects of temporal resolution in SPT for bidimensional movement (e.g., on cell membranes) considering all the steps of the analysis workflow.

First, we perform MSD analysis and diffusion coefficient estimation using exact simulated trajectories; then we perform the tracking analysis on exact simulated positions; finally, we carry out detection, localization and tracking on simulated movies. In our empirical investigation, we used the uTrack software, the most cited and used algorithm for SPT in biological applications to our knowledge [9, 23]. It was developed and is available in MatLab, but its tracking algorithm is also implemented in ImageJ and is available in the TrackMate plugin [24]. We investigated temporal resolution effects at different diffusivities, SNRs and particle densities. We show that, in practical situations, the impacts of other aspects besides localization must be considered, e.g. those related to the tracking step. The different investigated parameters can cause shifts and broadening on the obtained distribution of diffusivities, compromising the accuracy of the results. The effects of the different parameters in the different steps of the analysis interacts in non-trivial ways, highlighting the importance of analysing them one at a time.

For comparison with the computational results, we also performed SPT experiments. We labelled the p75^NTR^ receptor with a fluorescent dye and visualized it at the single molecule level on the membrane of living cells by total internal reflection (TIRF) microscopy. Thanks to previous investigations of simulations, we used the best temporal resolutions for our experimental conditions. From the experimental results, we could confirm a temporal range for obtaining comparable and reliable diffusivity distributions.

Thanks to the set of codes developed in the study and made freely available, optimal temporal resolution conditions can be found in different types of single particle tracking applications, following a procedure similar to the one shown by us.

## Results and discussion

### Simulation conditions

We simulated tracks of particles diffusing with Brownian motion on a square two-dimensional area usually 16 × 16 (= 256) μm^2^ in size; we did not consider other kinds of motions because we wanted to have a clear comparison with the expected diffusivity distributions. We set diffusion coefficients D_S_ at 0.1, 0.5, 1 or 2 μ m^2^/s; this range includes measured diffusion coefficients of membrane proteins, especially receptors, but also of lipids [25–30]; we did not use lower diffusivities because the expected influences of temporal resolution would be negligible. In order to keep the mean density of particles constant and to have a complete simulated trajectory for each particle, we set reflective boundaries for our simulated area. The temporal simulation step was 1 ms. On the obtained results, we simulated different temporal resolutions, as better explained in each of the following sections, using resolutions from 1 to 150 ms (therefore covering a rather exhaustive range of resolutions used in different kinds of single particle tracking applications [31–34]).

### Analysis of exact simulated trajectories

**Fig. 1:**
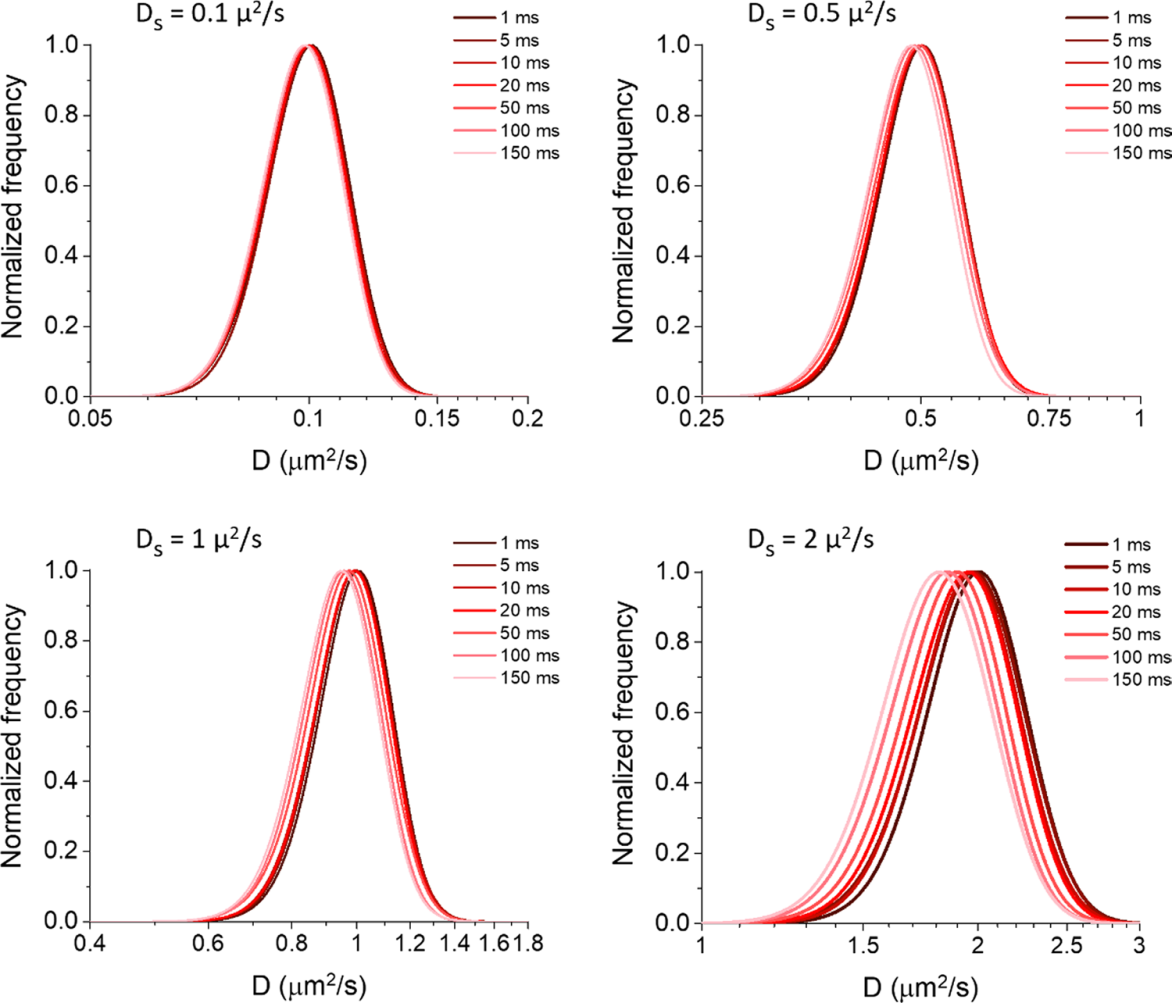
Diffusivity distributions determined starting from exact tracks. Tracks simulated using different diffusion coefficients D_S_ (as reported at the top of each graph) and time steps Δt (as reported in the legends) were analysed to extract the reported diffusivity (D) distributions as calculated from the first two points of the MSD function. Simulations were performed on a square area of 256 μm^2^ with reflective boundaries at a density of 0.3 particles/μm^2^. The different temporal resolutions were obtained by appropriate sampling on the same simulated tracks, stopping at 500 time steps in each case. 385 tracks from 5 different simulations were analysed for each case

In order to control if there were any effects of time resolution caused already by our simulation conditions (and to have a starting point to be compared in the following steps), we checked the diffusion coefficient distributions extracted from the exact trajectories. To obtain them, we sampled the simulated tracks using different temporal sampling intervals (Δt = 1, 5, 10, 20, 50, 100, 150 ms), with 500 total time steps for each case. On these sampled tracks, we performed the MSD analysis and estimated the short-time diffusion coefficient D from the first two points (time lag = 1 and 2), as previously reported [15, 18, 35]: importantly, this short-lag D can be used independently of the kind of motion, minimizing the impact of eventual drifted or confined motions, and is more sensitive for detecting changes in diffusivities [36, 37]. We observed a tendency towards an underestimation of diffusivity, assessed by the peak of its distribution D_peak_, at longer Δt, with a more evident effect at faster diffusivities (Fig. 1). We hypothesized that this phenomenon was only due to the confined motion caused by the reflective borders in our conditions, as already observed by others [38, 39]. To verify this point, we performed the same analysis on tracks simulated similarly on a surface with periodic boundaries (Fig. S1A). In this case, we observed no shift of the distribution of D, but a broadening at longer Δt. We also observed that the shift of the distribution observed in Fig. 1 becomes more pronounced for smaller areas (Fig. S1B). The broadening observed in Fig. S1A is due to the different trajectory lengths (i.e., number of spatio-temporal points that make up the track) in the case of periodic boundaries: at longer Δt, a particle can typically reach the boundaries (with interruption of its track) after a lower number of time steps, increasing the uncertainties on the estimated D. We further confirmed this point by simulating and analysing tracks on a surface without any borders, at a fixed Δt of 50 ms, by changing the number of points included in the track (n = 100, 250, 500, 1000, 2500; Fig. S1C). We observed the broadening of the D distribution at lower numbers of points, with a slight apparent shift of the peak at 250 and 100 numbers of points. The latter is caused by the semilogarithmic scale we use for the distribution function (as done in previous works [15, 26, 27]), and by the fact that when the standard deviation of the distribution in linear scale is close to or higher than the value of its average (situation that occurs at the lowest numbers of points in the tracks), this shifts the peak in the semilogarithmic distribution at a higher value (see Supplementary Note 1). It is interesting to notice that neither an often used approximation for the MSD of a confined motion nor a simple model developed by us could reproduce exactly the results retrieved from the simulation, the former by largely overestimating the expected short-lag D, and the latter by underestimating it at the low simulated D_S_ (see the following section and Supplementary Note 2). Instead, Fig. 1 allows having a quantitative measure of the effects of the reflective borders (set for all the simulations reported in the following sections) on the estimation of the diffusion coefficient in our conditions; we will show that this shift is typically smaller than others observed in the following, so it can be facilely considered in the interpretation of the results.

### Tracking on exact simulated positions

**Fig. 2:**
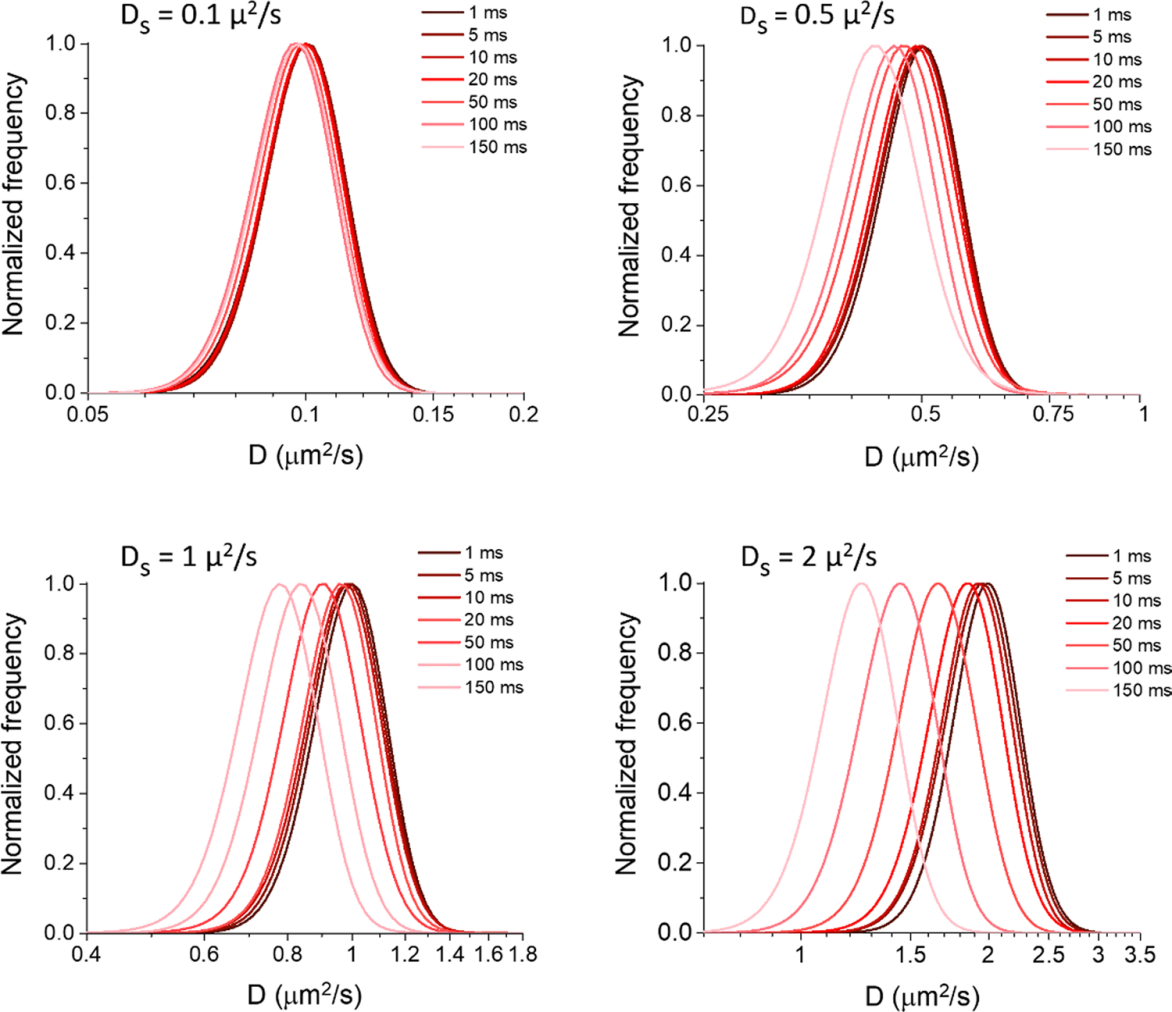
Measurement of diffusivity distributions after tracking on exact positions. Positions extracted from the same simulated tracks used for Fig. 1 were used as input in the tracking algorithm to measure the reported diffusivity (D) distributions. See Fig. 1 for additional details

**Fig. 3:**
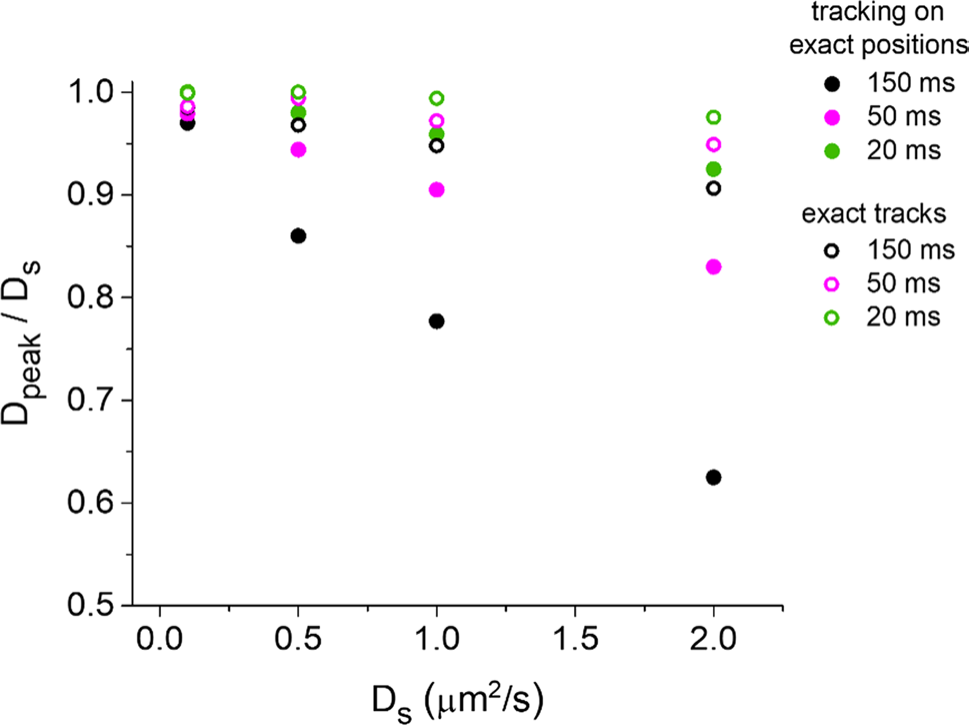
Comparison of the diffusivity underestimation effects between analysis of simulated tracks and tracking on simulated positions. The graph reports the ratio between the peak value obtained in the diffusivity distribution estimated after the analysis (D_peak_) and the corresponding simulated value (D_s_) as a function of the latter. Results are reported for three illustrative Δt (as reported in the legend), in the case of analysis on the exact simulated tracks (empty circles) and in the case of tracking on exact simulated positions (filled circles), and are extracted from the data in Figs. 1 and 2

In order to explore the possible effects of the tracking process on the D estimation, for each simulation step we considered only the positions of the whole set of spots generating the simulated trajectories sampled at various intervals Δt as specified above. We used these exact positions as input for the tracking algorithm, as if they were spots detected from an experimental movie. We observed a further underestimation of D, the more important the longer the Δt and/or the bigger the D_S_ (Fig. 2); the effect is bigger than the one caused by confinement in our simulations, as highlighted by Fig. 3 where we directly compare D_peak_ in the two cases considered until now. This additional effect has to be caused by inaccuracies in the tracking process.

In qualitative terms, it is known that there can be more tracking errors in SPT as the particle density increases, because of the ambiguity in the assignment of particle correspondences [8, 11, 22]. However, the effects are not quantitatively clear (concerning e.g. the estimation of a parameter such as the diffusivity) and predictable and have a dependency on the used tracking software [8]. Jaqaman et al*.* observed that, as the particle density increases, so do the following quantities: the fraction of particles with nearest neighbours closer than twice their average frame-to-frame displacement; the average number of potential assignments per particle; the fraction of particles with more than one potential assignments; instead, the average nearest neighbour distance decreases [9].

Our data highlight that, rather than a simple density intended as number of particles per unit area (kept fixed in the simulations in Fig. 2), the focus should be on a scaled “density” relevant for SPT. The crucial parameter to be considered is the ratio between the mean frame-to-frame displacement $\sqrt{4{D}_{S}\Delta t}$ and the mean nearest neighbour distance that scales with the particle density $\rho$ as $1/\sqrt{\rho }$ (in 2D). As this ratio increases, tracking errors increase [8, 9, 19, 22]; indeed, the possible assignments per particle are mainly established using motion information (this is typically true in tracking algorithms [8, 9]) and as the frame-to-frame displacement increases, the area in which possible connections are searched grows: the higher the density, the more the possible assignments in this area [9]. This kind of effects causes the underestimation of D observed in Fig. 2, due to a preference for shorter connections in the case of ambiguities, such as crossing trajectories. We also verified that the underestimation was less severe at lower particle densities (Fig. S2). Moreover, we checked that the observed underestimation depends only on the product of D_S_ and Δt, since the particle displacement depends solely on this product. Indeed, Fig. S3 shows that the underestimation of D is comparable for equal D_S_ x Δt, and it is greater for higher values of this product, as expected. Small differences within the groups are within the uncertainties, and follow roughly those found also in the D_peak_ obtained from exact simulated trajectories; they are therefore attributable to intrinsic variability in the simulations.

We tried to explain theoretically our observations; we found indeed that the underestimation of D can be linked to the errors in tracking described above, but we found quantitative discrepancies between theory and results (see Supplementary Note 2 and Fig. S4); this highlights the difficulty of integrating all the effects (also related to the functioning of the specific tracking software) into a theoretical description and the importance of an empirical investigation.

It is important to be aware of the impact of the interplay amongst particle density, diffusivity and temporal resolution in SPT: e.g., some works showed that only diffusivities estimated in the case of motions different than pure Brownian, such as confined or hop diffusion, can be affected by temporal resolution in single particle tracking [38, 39], but these kinds of evaluations were performed on simulated tracks (like we do in Sect. 1) and do not take into consideration the tracking process involved in the analysis of experimental data. So, an effect like the one observed in Fig. 3 may be misinterpreted as the presence of these other kinds of motion if the effects due to the tracking process itself on D estimation are not known.

### Detection and tracking on simulated movies

In the subsequent step, we also introduced the detection phase in the analysis. First, we obtained from the simulated trajectories the positions of all the particles for each simulation time step (1 ms). Then we simulated movies, in which each frame image was created through a pixelization of intensity spots positioned at the determined positions with a Gaussian point spread function, adding constant intensity background and noise to each pixel (as better described in the Materials and Methods section). We tuned the noise parameters to create two different conditions of SNRs for each case (see Materials and Methods, and Figs. S5 and S6). The different time resolutions Δt were obtained by summing sets of consecutive frame images; in this way, we also simulated the blurring of the spots due to particles motion during the experimental signal acquisition. Finally, we performed the whole single-particle-tracking analysis on the obtained movies, including both detection and tracking steps. From the reconstructed trajectories, we extracted the diffusion coefficient distribution as discussed in the previous paragraphs.

**Fig. 4:**
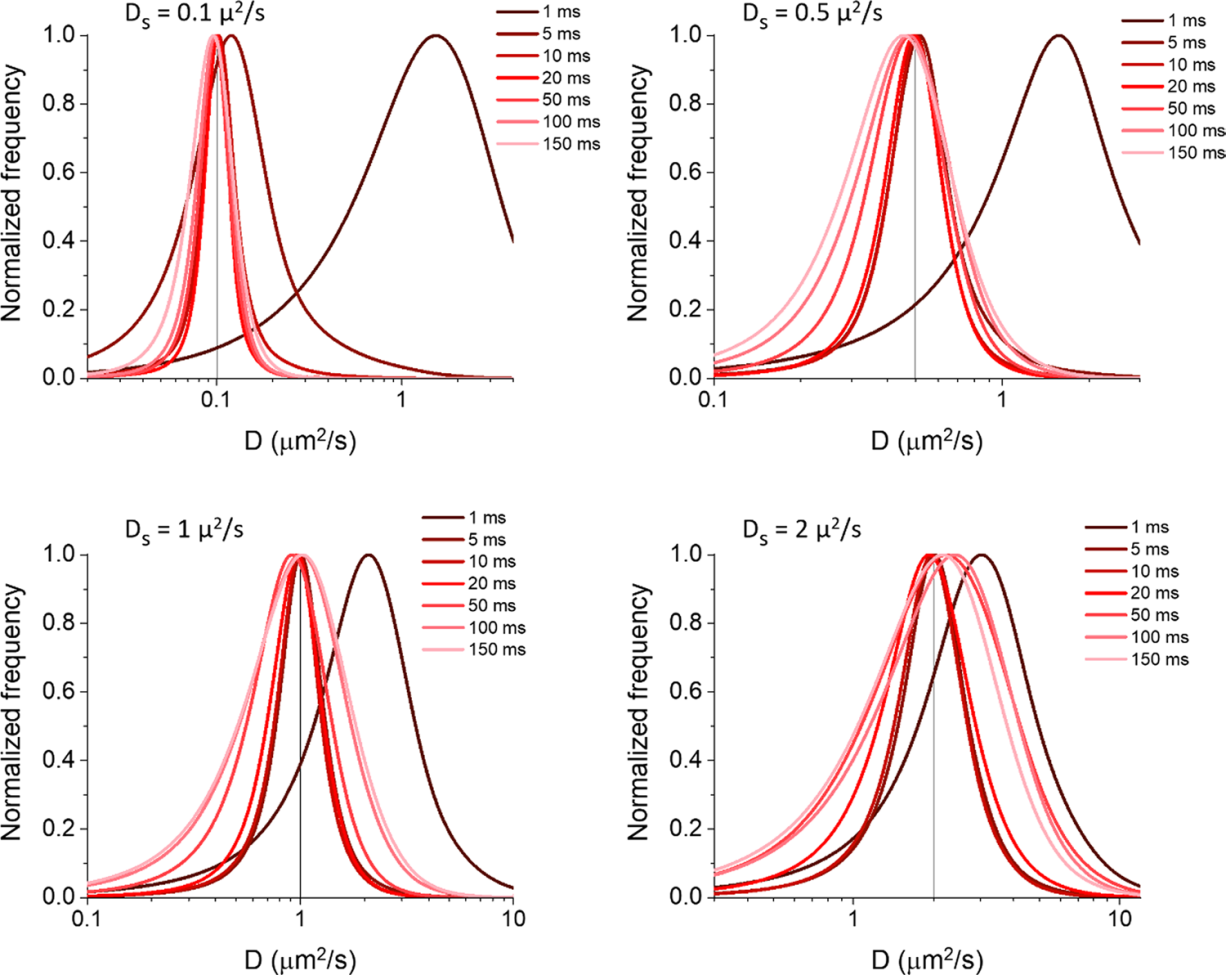
Measurement of diffusivity distributions after detection and tracking on the simulated movies with the higher used signal-to-noise ratio (SNR). The spot positions used for Fig. 2 (see the captions of Figs. 1, 2 for additional details; the used D_S_, reported within each graph, are indicated by the vertical line) were used to create simulated movies with the background and noise used in the case of higher SNR (example of images in Fig. S5). The movies were processed through spot detection and tracking to measure the reported diffusivity (D) distributions

**Fig. 5:**
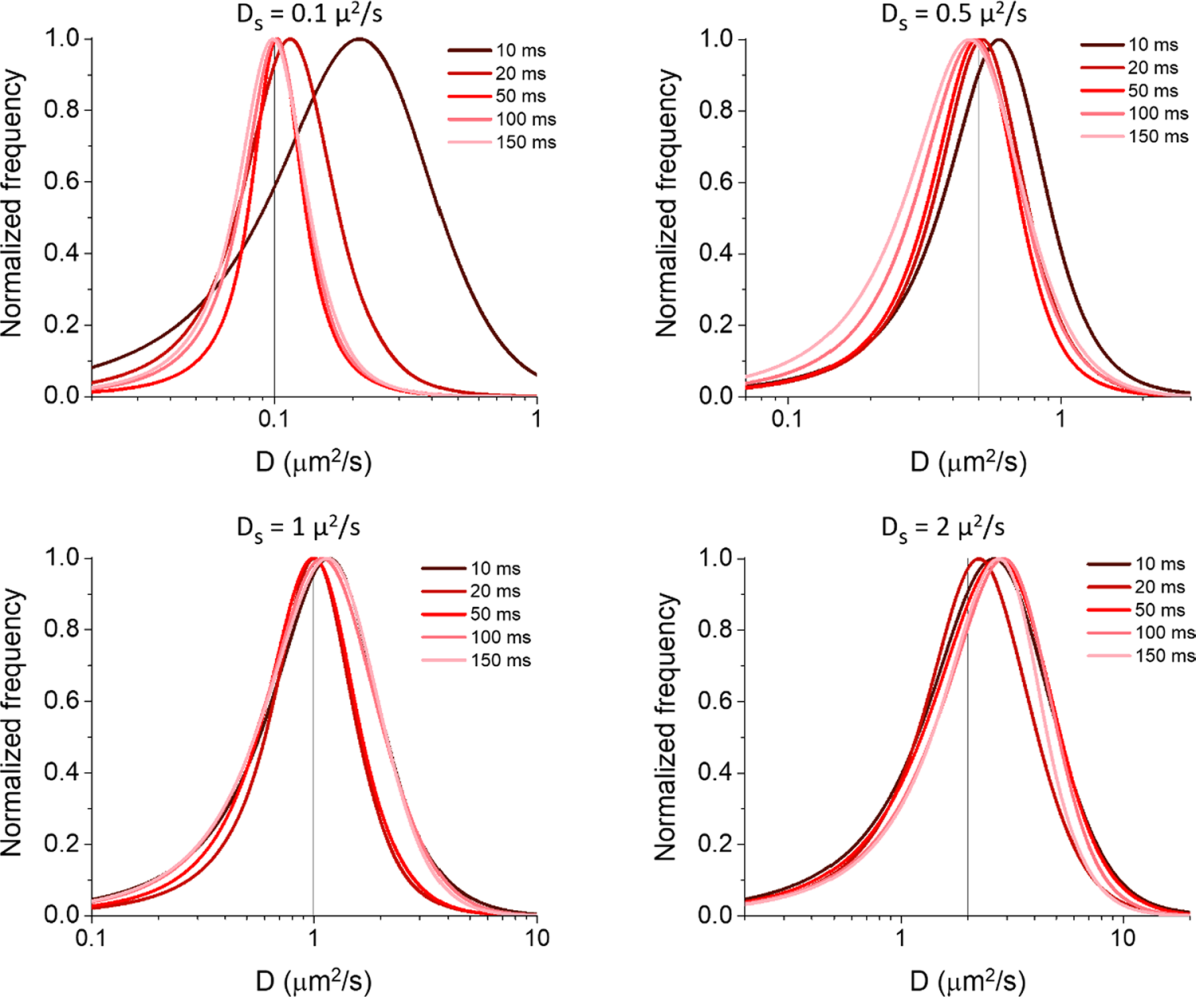
Measurement of diffusivity distributions after detection and tracking on the movies simulated with the lower considered signal-to-noise ratio (SNR). The same spot positions used for Figs. 2 and 4 (see the captions of Figs. 1 and 2 for additional details; the used D_S_, reported inside each graph, are indicated by the vertical line) were used to create simulated movies with the background and noise used in the case of lower SNR (example of images in Fig. S6). The movies were processed through spot detection and tracking to measure the reported diffusivity (D) distributions

**Fig. 6:**
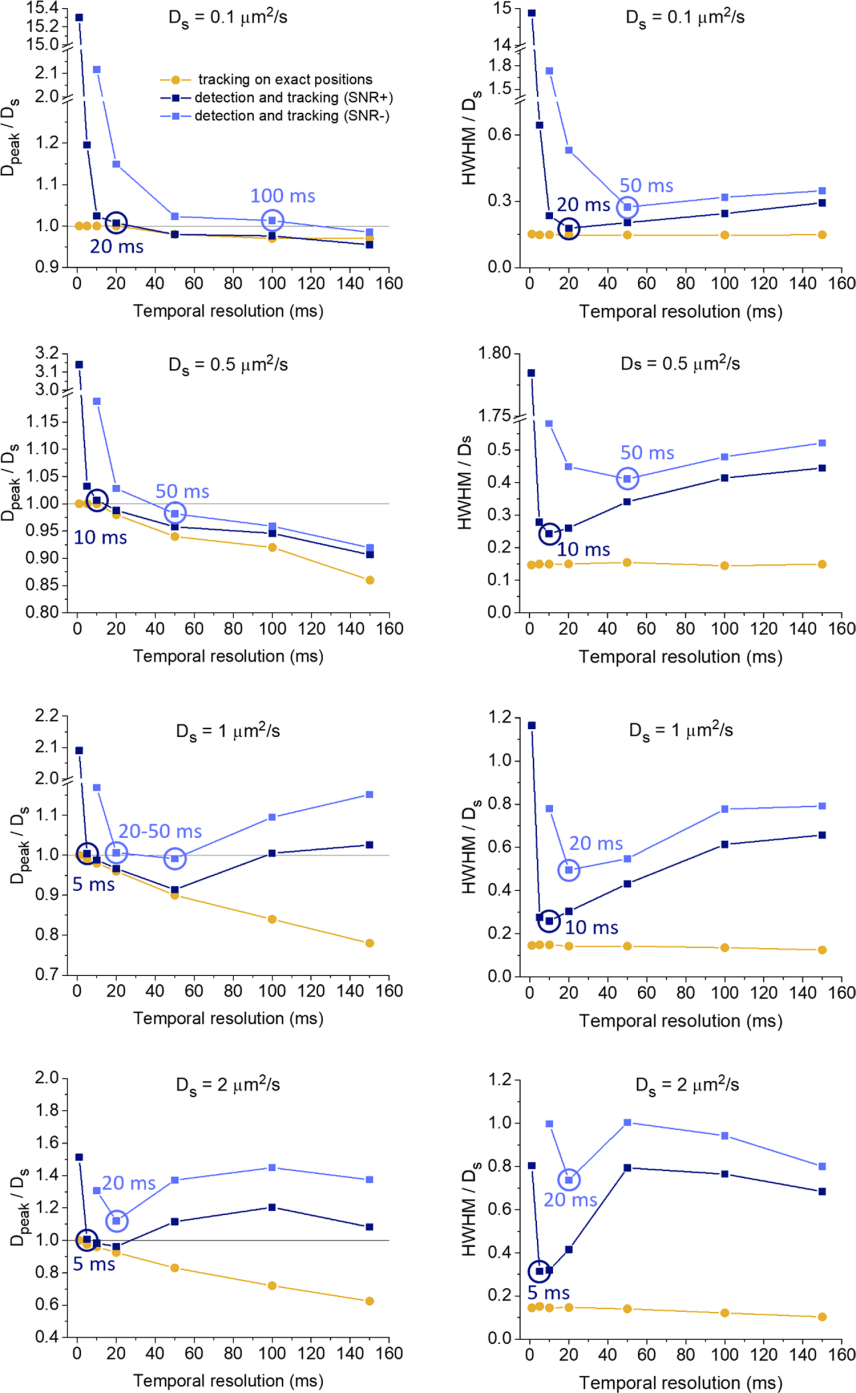
The graphs in the left column report the ratio between the peak value obtained in the diffusivity distribution estimated after the analysis (D_peak_) and the corresponding simulated value (D_s_) as a function of the temporal resolution. The graphs in the right column report the ratio between the half width half maximum (HWHM) of the estimated diffusivity distributions and D_s_ as a function of the temporal resolution. Results are shown for the case of tracking performed on exact simulated positions (yellow dots, corresponding to data in Fig. 2), and the cases of detection and tracking of simulated movies at higher (dark blue squares, SNR+, corresponding to data in Fig. 4 and images in Fig. S5) and lower (light blue squares, SNR−, corresponding to data in Fig. 5 and images in Fig. S6) signal-to-noise ratio. For the results from simulated movies, the data corresponding to the D_peak_ most similar to D_s_, and the smaller HWHM, are highlighted with circles in the corresponding colours

Figures 4 and 5 show the obtained distributions (at the lower simulated SNR for Δt = 1 and 5 ms, the spots could not be detected, so only the results for higher values of Δt were reported in Fig. 5), and in Fig. 6 we summarize the results, reporting also the data obtained starting from exact simulated positions for a direct comparison. In this last case, the estimate of D is equal to its true value at the shortest Δt and then decreases, the more and at shorter Δt the higher D_S_. Instead, when performing both detection and tracking, D is overestimated at the shortest Δt. Then, as Δt increases, D_peak_ first decreases approaching the true value, then for the lowest D_S_ it continues to decrease following the results of tracking on simulated exact spot positions, while at higher D_S_ the trend is reversed, with a further increase in D_peak_. The half width at half maximum (HWHM) of the D distribution peak is larger at shorter Δt, then, as Δt increases, it decreases toward a minimum and then increases again; at the highest diffusivities, the HWHM decreases again for even longer Δt. We have reported the HWHM/D_S_, to highlight conditions in which this quantity is close to or greater than 1 causing an important shift in D_peak_ (see Supplementary Notes 1 and 2). Minor changes on the HWHM were instead observed in the case of tracking on exact positions.

Introducing the detection phase, the effects connected to both static and dynamic localization uncertainty emerge, and temporal resolution affects both of them (see also Supplementary Discussion). Indeed, as Δt increases, static uncertainty decreases, due to better static SNR; at the same time, dynamic uncertainty increases, due to the blurring created by particle motion during the acquisition time of each frame (so worse dynamic SNR) [40]. This increasing impact of dynamic uncertainty is likely responsible for the reversal of the trend in the shift in the distribution of D for higher D_S_ compared to the case of tracking on exact positions. Indeed, higher localization uncertainties lead to higher uncertainties and broader distributions for D, causing a positive shift for D_peak_ due to the impact of the standard deviation of the distribution on the peak position in logarithmic scale, as noted above (see also Supplementary Note 1). More importantly, when the SNR is too low, a significant fraction of the spots cannot be detected and this will affect the tracking step: if a spot in a track cannot be detected, there can be an erroneous link to a spot of a different track, likely more distant (depending on temporal resolution, particle density and diffusivity), and so the diffusivity can be overestimated. Also motion blurring can cause missed or erroneous detections (see Fig. S7): indeed, it alters the shape of the spots from the Gaussian profile used in most localization algorithms; on one hand, motion blurring has a greater effect on the detection of faster-moving spots and can therefore cause a bias towards an underestimation of the diffusivity, since faster tracks cannot be reconstructed correctly; on the other hand, similarly to the case of low SNR, if some of the spots are missing, connections longer than the true one can cause an overestimation of the diffusivity.

We highlighted the best conditions in each graph considering that it is desirable to have a peak value as close as possible to the true one and narrow distributions (for more accurate estimations and better distinction of possible different diffusive states and heterogeneities); in most cases, these conditions are the same or almost the same when considering the HWHM and the position of the peak value.

In general, higher static SNR allows using a better temporal resolution (lower Δt). Moreover, in both SNR conditions, as D increases, a lower Δt is required, due to the effects of dynamic uncertainties.

### Single particle tracking on p75^NTR^ receptors

**Fig. 7:**
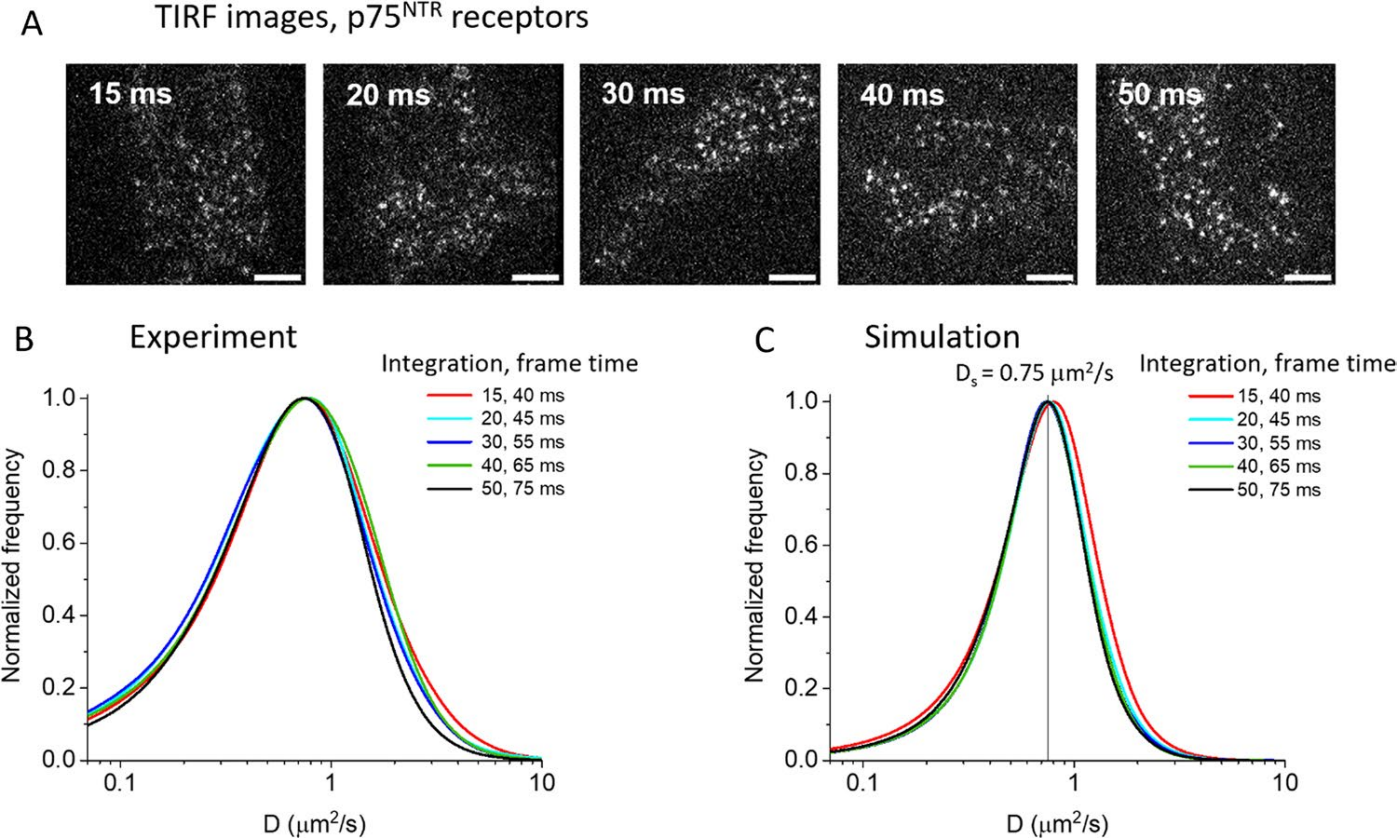
Single particle tracking experiment on p75^NTR^ receptors fluorescently labelled on the membrane of living SK-N-BE(2) neuroblastoma cells and comparison with simulated results. **A** Experimental images from the movies recorded by TIRF microscopy; on each image we report the integration time; frame time was 25 ms longer due to readout time. Images are shown in the same fixed grayscale; scale bar: 5 μm. **B** Corresponding measured diffusivity distributions. Mean particle density was 0.3 µm^−2^. Data were obtained from 2180 to 3050 tracks for each case. **C** Diffusivity distribution derived from detection, tracking, and MSD analysis on simulated movies with integration, frame times as written in the legend. Particle density was 0.3 µm^−2^, D_S_ was 0.75 μm^2^/s (equal to D_P_ for all curves but the red one for which it is 0.8 μm^2^/s). 385 tracks from 5 different simulations were used for each case. In both **B** and **C** 130-frames movies were analysed for each temporal resolution. We calculated the uncertainties in the distributions [27] for both the experimental and simulation cases; some examples are reported in Fig. S9

In order to test the simulation results with the conditions of real experiments, we performed single particle tracking on fluorescently labelled p75^NTR^ receptors observed by TIRF microscopy on the membrane of living cells (Fig. 7). Our experimental conditions of SNR are similar to those used in the simulations with lower SNR; moreover, diffusivities reported in the literature for this receptor in the cell line we used (neuroblastoma SK-N-BE(2)) are around 0.7 μm^2^/s [26]. So, from our simulation results (Fig. 6), optimal time resolution conditions could be approximately between ~ 20 and ~ 50 ms. We acquired experimental movies using image integration times of 15, 20, 30, 40, 50 ms; in our setup these correspond to frame times of 40, 45, 55, 65, 75 ms (due to the EMCCD readout time of 25 ms). Shorter frame times were not allowed due to limitations in static SNR, and longer frame times resulted in too much photobleaching. We performed the detection and tracking analysis and then extracted the histograms of diffusivities as in the previous paragraphs (Fig. 7B). We obtained comparable distributions for the applied temporal resolutions with a peak value of ~ 0.75 μm^2^/s. We performed simulations more similar to the experiments, with integration and frame times having the same experimental values and setting D_S_ at 0.75 μm^2^/s (Fig. 7C). We observed a slight shift compared to the true value only for the lowest integration time of 15 ms (likely due to a low SNR); in all other cases, the distributions are comparable and the peak is located at the value set in the simulations. Therefore, we can confirm that we identified a range of temporal resolutions allowing a reliable diffusivity estimation in our experimental conditions. In order to check if the Brownian dynamics approximation we used in the simulation agreed with the experimental data, we calculated the two-dimensional distributions of the anomalous coefficient (see Materials and Methods section) versus the diffusion coefficient in both cases. The distributions had a similar shape for both experiment and simulation (with broader distributions in the case of the experiment, as expected); the peak of the anomalous coefficient was close to 1, indicating the prevalence of pure Brownian motion (an example is shown in Fig. S8, but the distributions look very similar also at higher and lower integration times).

## Conclusions

We performed a systematic investigation of the effect of temporal resolution in single particle tracking, quantifying its influence on position and width of the resulting distributions for diffusivities in the case of pure 2D Brownian diffusion with a single diffusivity D_S_. Despite the presence of other types of possible motions in SPT applications, the Brownian one remains one of those of main interest, often considered first in the development and evaluation of SPT software and analysis methods [8, 21, 41, 42].

We studied the impact of temporal resolution in the different phases of the analysis, using simulations of trajectories and movies, a popular tracking software for the analysis, and introducing one step at a time.

Using exact tracks, we observed the effect of confinement on short-lag D, caused by the boundaries of a limited area available for particles; we quantified the caused underestimation of D depending on the values of diffusivity and time resolution, the kind of boundaries and the area dimensions. Using exact simulated positions to apply the tracking analysis, we showed that tracking errors tend to cause a greater underestimation of D, the bigger the product between particle density, Δt and D (i.e. the square of the ratio between average frame-to-frame step length and average interparticle distance). Introducing the complete analysis (detection and tracking) on simulated movies, we also included the impact of localization uncertainty (the higher the lower the SNR or the broader the spot blurring caused by motion); we observed that it causes a broadening of the D distribution, and this causes an apparent overestimation of its peak position at lower time resolutions and consequently lower SNR (especially when plotted, as usually done, in a logarithmic scale); moreover, it also causes shifting of the D distribution that combines with previous underestimation effects and becomes prevalent for higher values of D and Δt. In each studied situation, we identified the best temporal resolution conditions, i.e. the ones producing the narrowest distributions of D and with the peak the closest to the true value, highlighting the dependency on the SNR and the diffusivity.

The optimal conditions were applied in an experimental case, i.e. single particle tracking of p75^NTR^ receptors visualized by TIRF microscopy on the membrane of living cells, in which we confirmed the reliability of results in the identified range of temporal resolutions.

As we showed, the use of simulations allows for identifying an optimal range of temporal resolutions for the experiments, anticipating if the obtainable temporal resolutions in a setup would impact the expected results (and therefore, deciding if the experiments should be performed at all or how they can be improved), and in general avoiding performing more experiments at different temporal resolutions (being therefore a faster and more cost-effective procedure).

Our study and the implementation of the algorithms for repeating it in other situations, which we are making available, will help to increase the awareness of temporal resolution effects on SPT and on the reliability and accuracy of extracted quantities. Indeed, SPT is nowadays a quite popular technique, and with the increasing of its applications, the need for studies highlighting possible experimental and analytical pitfalls and misleading interpretations of results increases as well [21, 43–45]. For example, some studies in this direction investigated the impact of the probe on the accuracy of SPT data, showing that some of the commonly used ones actually alter the dynamics of the molecules of interest [43, 45]. Temporal resolution is a critical parameter for SPT, which determines the observability of a process, such as important biological mechanisms [46], and is crucially connected to spatial resolution: a compromise between the two quantities is required [19]. In turn, spatiotemporal resolutions are connected to several experimental factors, from the choice of the label (with its brightness and photostability properties) to the performance of the setup and the selection of excitation and detection parameters [1, 10, 47]. Therefore, it is pivotal to have full awareness of the effects of the ensemble of chosen conditions. Our kind of approach could be used to test other experimental situations in the different contexts where single particle tracking is applied, and could be extended to other tracking software programs. Indeed, in the field of SPT, different programs continue to be developed and testing of their performance, especially by groups different from the developers, can provide more objective comparisons and indicate paths for more robust algorithms [8, 48, 49]. We hope that our work will contribute to a growing knowledge of the effects caused by the choice of all the parameters involved in SPT experiments and thus help more and more researchers for the accurate and robust designing, planning and analysis of these studies.

## Methods

### Simulations

Simulations of diffusing molecules were performed using homemade codes in Python. In most cases, we simulated points moving with Brownian motion within a square area with reflective boundaries (except for Fig. S1 where we also performed simulations on a surface without boundaries and with periodic boundaries). Assuming that the area boundaries are located at 0 and $L$ in the $x$-dimension, if the calculated $x_{c}$ coordinate for a point is greater than $L$, it will become $x_{c} - L$ in the case of periodic boundaries or $L {-} \left( {x_{c} - L} \right)$ in the case of reflective boundaries (and analogously for the y-dimension); if the calculated $x_{c}$ coordinate for a point is less than 0, it will become $x_{c} + L$ in the case of periodic boundaries or ${-}x_{c}$ in the case of reflective boundaries (and analogously for the y-dimension). Starting positions were distributed randomly with uniform probability in the area and the displacement for each simulation time step was calculated along x and y dimensions by a Gaussian distribution with mean 0 and standard deviation $\sqrt {2Ddt}$, where values of the diffusion coefficient D were varied as stated in the results and figures and the simulation temporal resolution $dt$ was set to 1 ms.

In the case of the analysis on exact trajectories, we used the tracks provided by the simulations for writing a.mat file (to be read in MATLAB) analogues to those produced by uTrack at the end of the tracking process. The different temporal sampling Δt was obtained by considering the positions in the track every each time step Δt (Δt = 1, 5, 10, 20, 50, 100, 150 ms).

In the case of analysis on exact positions, we extracted from the simulated tracks the positions of the set of points for each desired temporal sampling Δt. We produced a.mat file analogues to those produced by uTrack at the end of the detection process; this file could be used as input for the tracking step.

In the case of analysis involving detection and tracking on simulated movies, we extracted from the simulations the positions of the set of points for each simulated time step (1 ms). We generated movies creating intensity spots with a Gaussian point spread function (standard deviation along *x* and *y* 1.2 pixel = 192 nm) centered at the simulated positions, adding a constant background and a Gaussian noise. Values for integrated spot intensity, constant background and standard deviation of Gaussian noise were: 30 (± 20% with a Gaussian distribution, corresponding approximately to an average of 25 photons collected for each spot in 1 ms considering Poissonian uncertainty), 30 and 15 for the higher SNR and 40 (± 30%, as above, corresponding approximately to ~ 11 collected photons), 15 and 60 for the lower SNR, respectively. We used a pixelization with a pixel size of 160 nm. We summed consecutive images of the movie to obtain different temporal sampling. For simulating different integration time and frame time (due to simulation of the readout time), the images corresponding to the readout times were ignored. The obtained movies were processed through uTrack using both detection and tracking analyses.

### Cell culture, receptors expression and labelling

SK-N-BE(2) (ATCC® CRL-2271™) cell lines were cultured at 37 °C, 5% CO_2_ in DMEM/F-12 medium supplemented with 10% fetal bovine serum, 1% L-glutamine, 1% penicillin–streptomycin. 24 h before transfection, cells were seeded on WillCo-dish® Glass Bottom dishes (WillCo Wells). A1-tagged p75^NTR^ receptor construct (previously described [34]) was transfected without reconstruction of lentiviral particles by using Lipofectamine 2000 (Thermo Fisher Scientific) according to manufacturer’s instructions (as previously done [50, 51]). 5 h after the transfection, the expression was induced with 0.005 μg/ml of doxycycline added in the culture medium. After 24 h, receptors were labelled by using 2 μM Sfp Synthase, 10 mM MgCl_2_, 10 nM of Coenzyme A-conjugated form of Atto 565 dye in the culture medium, for 15 min at 37 °C; then cells were washed five times with PBS and immediately imaged under the TIRF microscope.

Purification of Sfp Synthase and production of Coenzyme A-dye were performed as previously described [50–52].

### Total internal reflection (TIRF) microscopy

Cells were imaged under a Leica DM6000 inverted microscope (Leica Microsystems) equipped with an epifluorescence module, DIC in transmission, Total internal reflection (TIRF)-AM module, four laser lines (405 nm, 488 nm, 561 nm, 635 nm), HCX PL APO 100X oil-immersion objective (NA = 1.47), electron-multiplying charge-coupled-device (EMCCD) camera (iXon Ultra 897, Andor) and incubator chamber to have 37 °C and 5% CO_2_ conditions for live-cell imaging; the resulting pixel dimensions were 160 µm.

For each acquisition, we selected a region of interest (ROI) of 159 × 147 pixels (corresponding to 25.44 × 23.52 μm^2^) including the membrane of a cell expressing labelled receptors, and we acquired 130-frame time series. We used different integration times (as specified in the results) and the frame time was 25 ms longer due to the readout time of the camera for the used ROI dimensions.

### Calculation of diffusion coefficient

On experimental movies, we first used the maximum intensity projection of a movie to identify the cell membrane (where observed spots were moving) and we applied a mask to exclude areas outside the cell. Masked movies were processed through detection and tracking. For tracking or detection plus tracking analysis we used the uTrack software (version 2.2.1 in MATLAB), due to its high popularity for single particle tracking in biological applications [9].

Detection and tracking parameters were optimized according to displacement and SNR conditions.

The parameters used for statistical tests for spot detection and the gap closing time window for tracking were optimized depending on the SNR conditions and by visual inspection of the detection and tracking results. The search radius lower and upper limits were scaled with the mean square displacement (depending on D and Δt values). The merging and splitting option was used only when the detection phase was used (so not in the case of tracking on exact positions). MSD analysis and calculation of the short-term diffusion coefficient D were performed in MATLAB as previously described [14, 15, 26, 51]. Briefly, we selected tracks composed at least of 6 detected spots and 9 frames including gaps. When the merging and splitting option was used, we extracted the subtrajectories separated by this kind of events. On the resulting tracks, we calculated the MSD and from the slope of the line passing through the first two points we obtained D. The distributions of D were calculated taking into account the uncertainties on D and weighting each trajectory with the number of frames when the spots have been detected; since typical standard errors estimated for D for each trajectory are usually bigger than the bin widths, the distribution results smooth, but each point in the curve representing it has an uncertainties; these have been calculated as previously reported [27] and some examples are shown in Fig. S9; note that, even if not shown in all the graphs for the sake of simplicity, the orders of magnitude for uncertainties for all the normalized distributions shown here are the same. Distributions were normalized to 1 at the peak. Peak positions D_peak_ and half width at half maximum (HWHM) were determined from the distribution of D in logarithmic scale, as in the graphs reported in the figures. Anomalous diffusion coefficient α for each trajectory and the combined distributions for D and α were calculated as in [53].

Theoretical estimates for D have been calculated with the help of Wolfram Mathematica 6.0.1.0.

### Supplementary Information

Additional file1 (DOCX 6328 kb)

## Data Availability

The datasets generated during and/or analysed during the current study are available from the corresponding author on reasonable request.

## Code availability

The codes developed in the study are available on GitHub at: https://github.com/chiaraSchirripaSpagnolo/SPT_D_vs_dt.
