# Supplementary material for: Impact of temporal resolution in single particle tracking analysis
Source: Discov Nano. 2024 May 9;19(1):87. doi: 10.1186/s11671-024-04029-1 (PMC11082114; doi:10.1186/s11671-024-04029-1)
Supplement: Supplementary file 1 — Additional file1 (DOCX 6328 kb) [file 11671_2024_4029_MOESM1_ESM.docx]

**Impact of temporal resolution in single particle tracking analysis**

**Chiara Schirripa Spagnolo^1*^, Stefano Luin^1,2*^**

^1^ NEST Laboratory, Scuola Normale Superiore, piazza San Silvestro 12, I-56127, Pisa, Italy

^2^ NEST Laboratory, Istituto Nanoscienze-CNR, piazza San Silvestro 12, I-56127, Pisa, Italy

* Corresponding authors: Chiara Schirripa Spagnolo [chiara.schirripaspagnolo@sns.it](mailto:chiara.schirripaspagnolo@sns.it); Stefano Luin [s.luin@sns.it](mailto:s.luin@sns.it)

# Supplementary Information

## Supplementary Figures


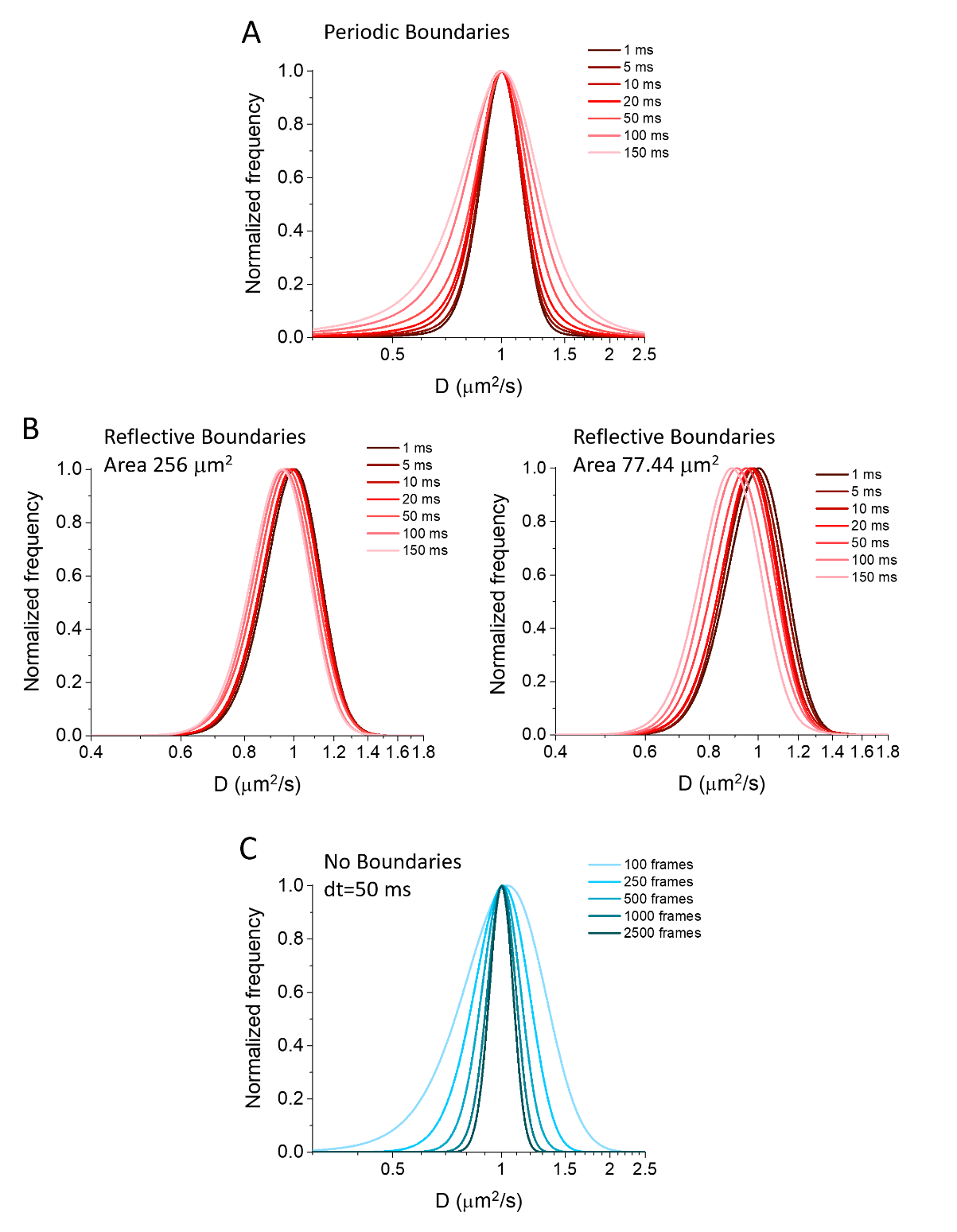


**Fig. S1** Effects of boundaries of the simulated surface on the estimation of diffusivity distributions determined from the first two points of the mean square displacement (MSD) for the exact tracks simulated with diffusivity D_S_=1 μm^2^/s. A) Simulations of diffusing particles were performed on a square area of 256 μm^2^ with periodic boundaries. A maximum duration of 500 time steps for the analysed tracks was used in each case. B) Simulations of diffusing particles were performed on a square area of 256 μm^2^ (left) and 77.44 μm^2^ (right) with reflective boundaries. A fixed duration of 500 time steps per analysed track was used in each case. C) Simulations of diffusing particles were performed on a surface without boundaries (an area of 256 μm^2^ was only used for initial positioning of the particles); temporal resolutions of analysed tracks was 50 ms, with variable number of frames as stated in the legend. For all panels: particle density was 0.3 particles/μm^2^; the tracks were simulated at a temporal resolution of 1 ms and following sampling on the same tracks was applied to obtain the different temporal resolutions; data were obtained for 5 independent repetitions of the simulations


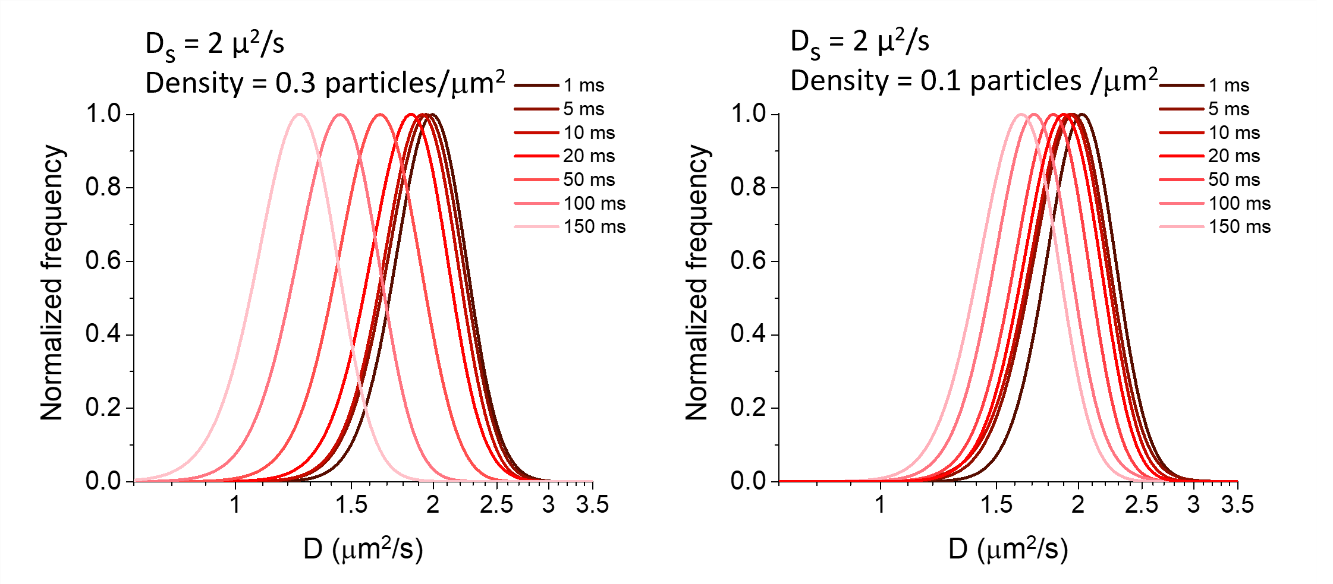


**Fig. S2** Effect of density on the estimation of diffusivity distribution. Positions extracted from simulated tracks (simulated diffusion coefficient value D_s_: 2 μm^2^/s) were used as input in the tracking algorithm to measure the reported diffusivity (D) distributions. Simulations were performed on a square area of 256 μm^2^ with reflective boundaries at a particle density of 0.3 particles/μm^2^ (left) and 0.1 particles/μm^2^ (right). The tracks were simulated at a temporal resolution of 1 ms; following sampling on the same tracks was applied to obtain the positions at different temporal resolutions, considering a fixed duration of 500 time steps in each case. 385 tracks from 5 different simulations were used for each case


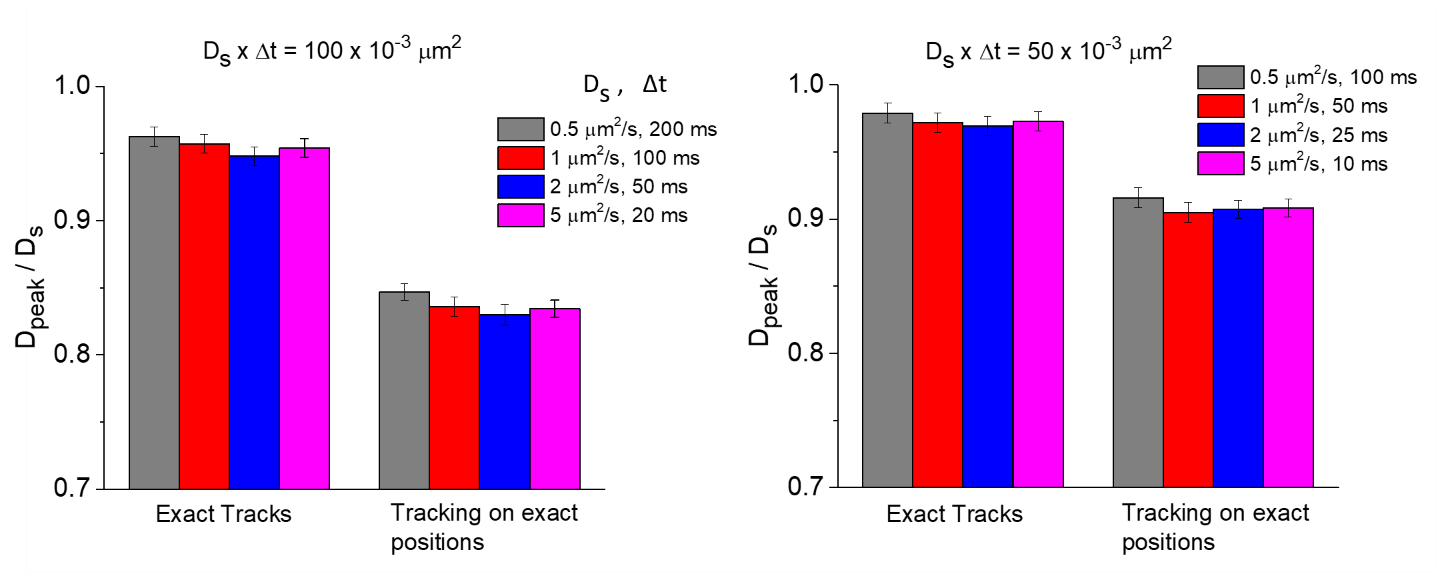


**Fig. S3** Comparison of accuracy in diffusivity estimation at fixed product of simulated diffusivity (D_s_) and temporal resolution (frame time, Δt), with constant spot density of 0.3 particles/μm^2^. Values of 50·10^-3^ μm^2^ and 100·10^-3^ μm^2^ for such product were considered. The y-axis reports the ratio between the peak value obtained in the diffusivity distribution (D_peak_) and D_s_. Results are shown for the case of analysis of simulated tracks (Exact tracks) and the case of analysis of tracks obtained from the tracking on simulated positions (Tracking on exact positions). Results are mean ± standard error, estimated by the half width at half maximum of the D distribution divided by the square root of the number of trajectories for its calculation

**Fig. S4** Comparison between theoretical (lines) and empirical (dots) results. Empirical data are the results of simulations analyses reproduced from Fig. 3, theoretical data are obtained as described in Supplementary Note 2. Continuous and dashed lines are obtained from two models for exact tracks confined in a 16x16 µm^2^ square area, and should be compared with the empty dots; dotted lines are obtained from a model for tracking on exact positions for free Brownian motion, and should be compared with the filled dots of corresponding color


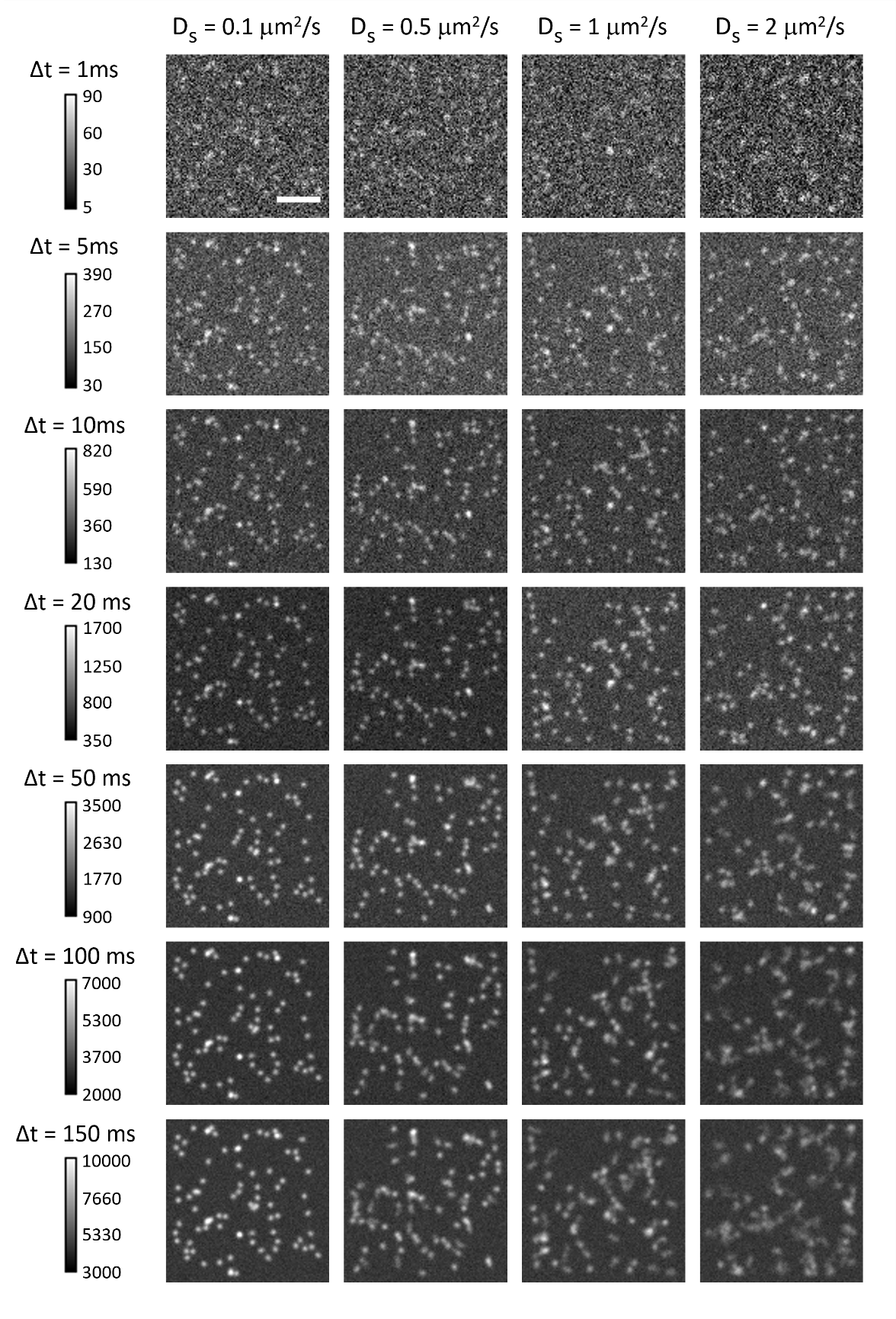


**Fig. S5** Example of images from the simulated movies at higher signal-to-noise ratio (SNR). Simulation parameters are specified in the Materials and Methods section. One image is reported for each condition of used values for simulated diffusion coefficient (D) and temporal resolution (Δt). Scale bar: 5 μm


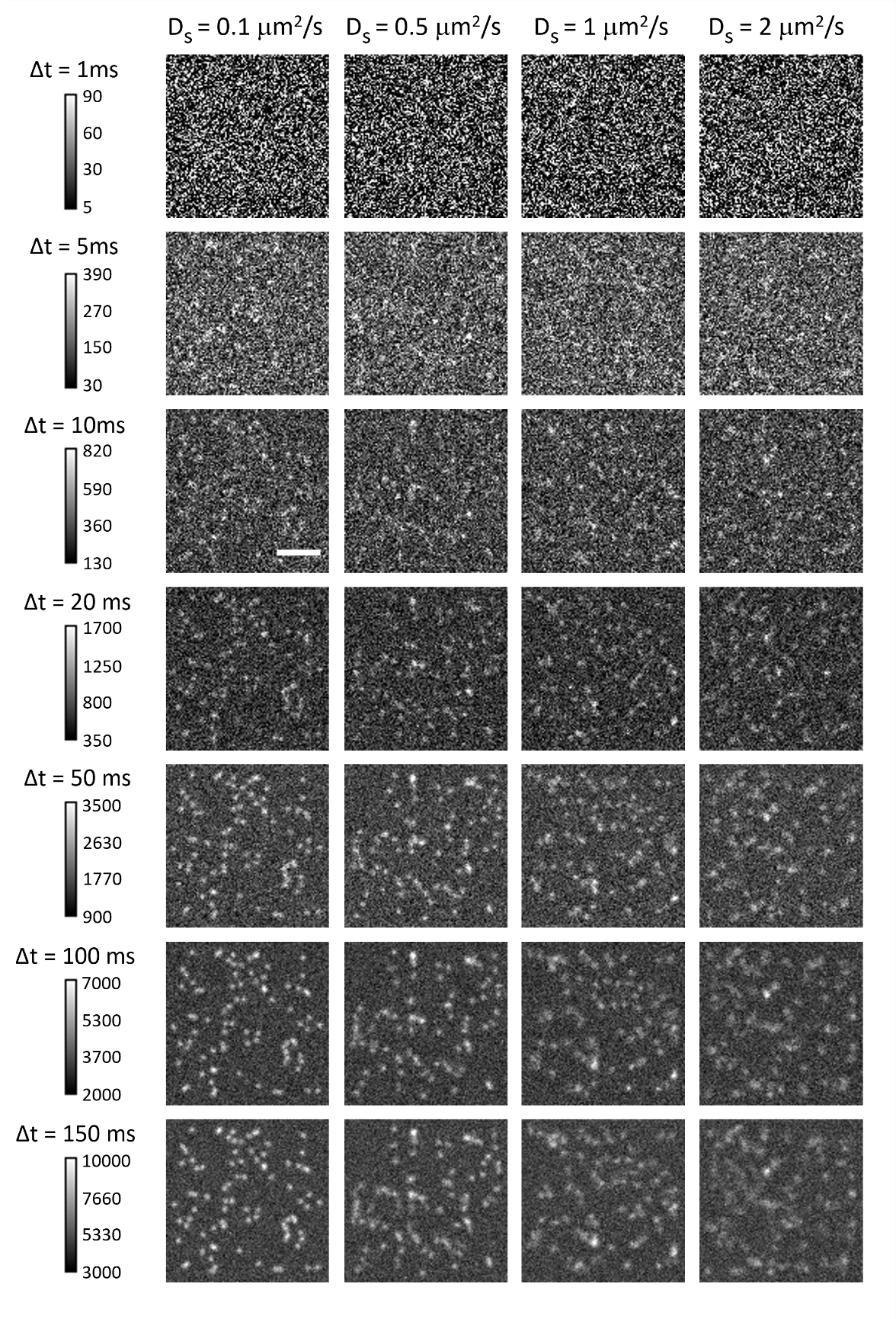


**Fig. S6** Example of images from the simulated movies at lower signal-to-noise ratio. Simulation parameters are specified in the Materials and Methods section. One image is reported for each condition of used values for simulated diffusion coefficient (D_S_) and temporal resolution (Δt). Scale bar: 5 μm

**
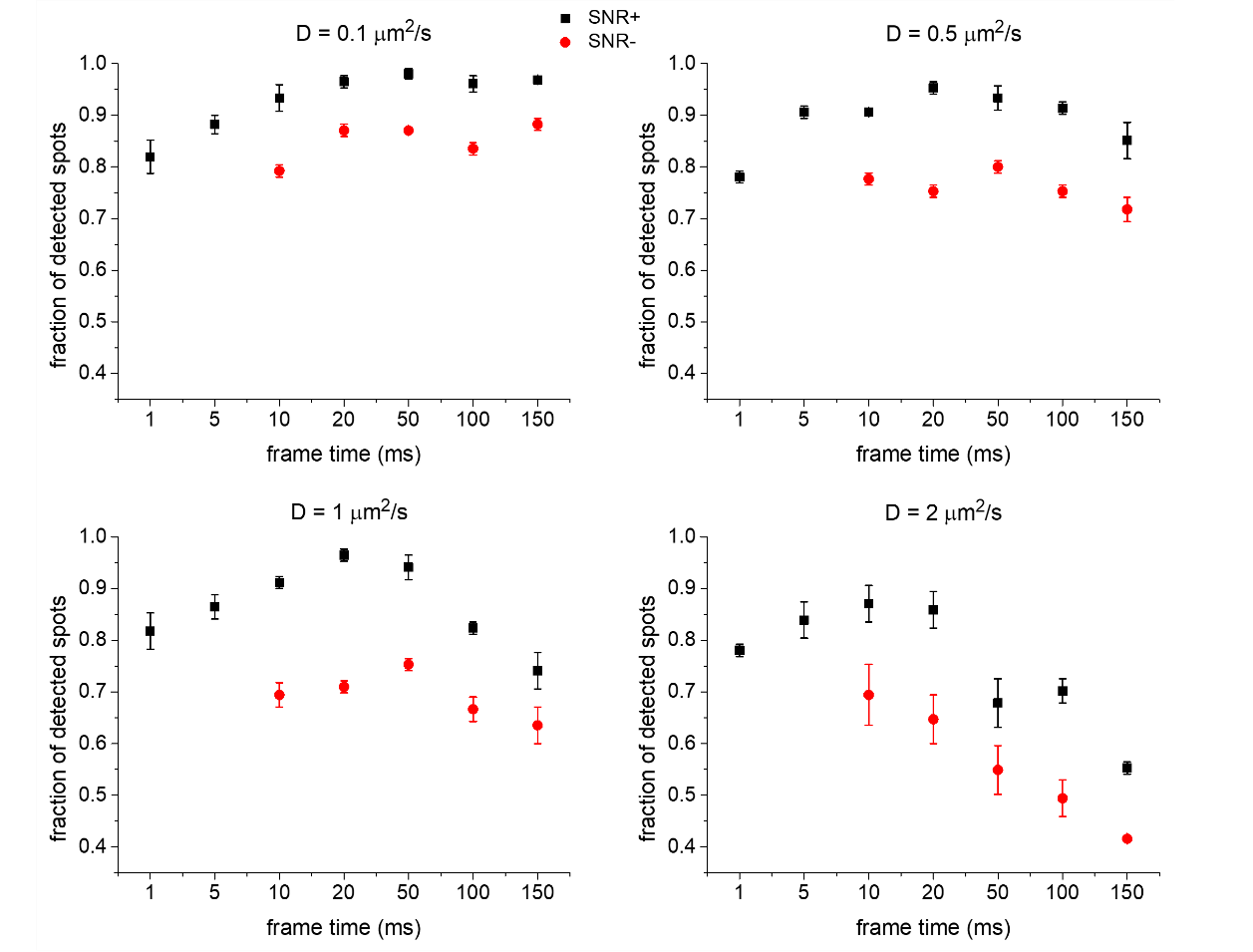
**

**Fig. S7** Fraction of detected spots over the total number of spots set in the simulations at different values of diffusivity and time resolution. Results are calculated on movies used in Figs. 4, 5, 6. Data are mean ± standard error of the mean


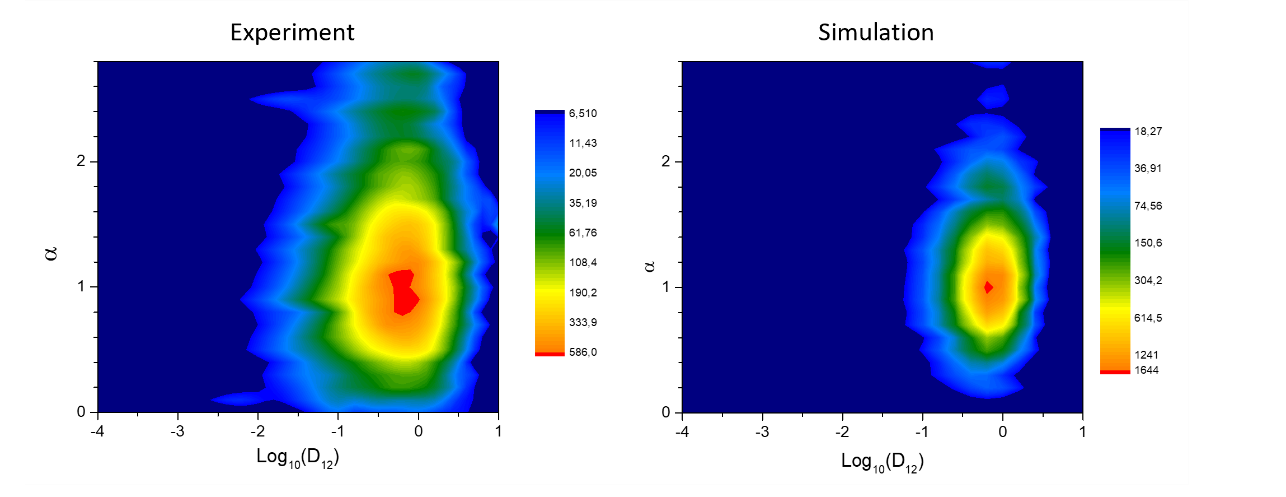


**Fig. S8** Examples of maps of the anomalous exponent (α) versus the diffusion coefficient (D_12_, reported in logarithmic scale). The integration time was 30 ms (and the frame time was 55 ms). Reported data correspond to experiments and simulations reported in Fig. 7


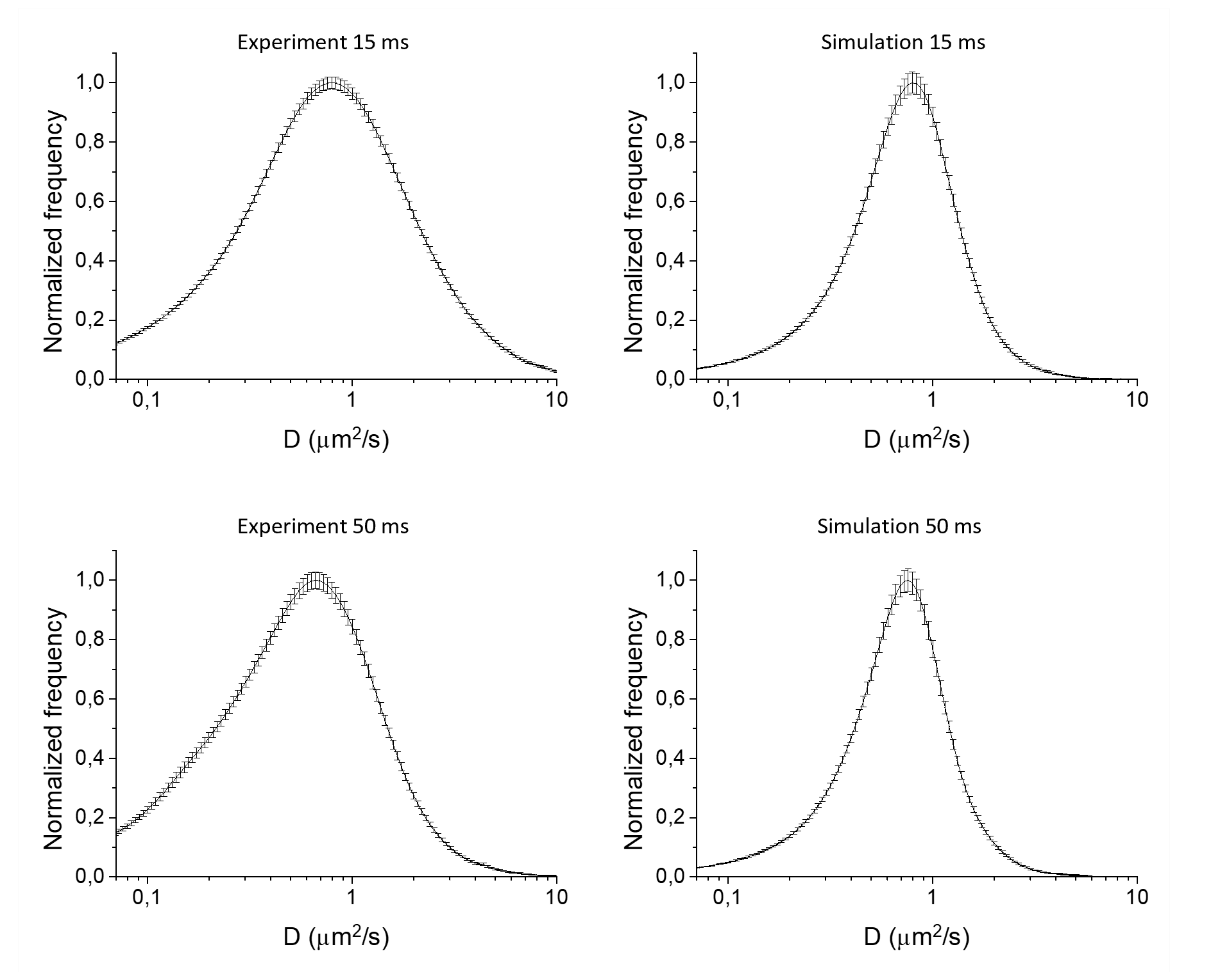


**Fig. S9** Examples of D distributions with error bars. Reported data are extracted from those in Fig. 7. Values of the uncertainties for normalized histograms do not depend on the chosen binning, or only very slightly; here we report a sparser binning than in Fig. 7 for the sake of clarity. The used integration time is shown at the top of each graph

## Supplementary Note 1

In order to understand how the width of the distribution $p$ of a parameter $x$ can affect the position of its peak when $x$ is reported in a logarithmic scale (*i.e.* $x={10}^{t}$, where $t$ is a parameter for the corresponding linear scale on the x-axis of the semilogarithmic graph, with distribution $\tilde{p}\left( t \right)$), we will consider the case of a Gaussian distribution centered on $X$ and with variance $\sigma^{2}$ for $x$:

$$p\left( x \right)dx\propto\exp\left( \frac{-\left( x-X \right)^{2}}{2\sigma^{2}} \right)dx, t=\log_{10} x \Rightarrow\tilde{p}\left( t \right)dt\propto\ln10\exp\left( \frac{-\left( {10}^{t}-X \right)^{2}}{2\sigma^{2}} \right){10}^{t}\mathrm{dt}$$

and $\tilde{p}\left( t \right)$ has a maximum when ${10}^{t}=\frac{X+\sqrt{X^{2}+4\sigma^{2}}}{2}$. Therefore, if $2\sigma$ is negligible with respect to $X$, the distribution in logarithmic scale is still peaked at a value corresponding to $X$; when the standard deviation of the distribution in linear scale is of the order of, or higher than, the value of its average, this shifts the peak in the semilogarithmic distribution at a higher value.

## Supplementary Note 2

The impact of localization errors (due to both static and dynamic uncertainties) on the Mean Square Displacement (MSD) function has been analysed especially for the case of pure Brownian motion. It has been reported that each of the two sources of errors causes the presence of a constant offset in the MSD function, positive for the case of static errors, negative for the case of dynamic errors [1, 2]. The MSD function takes the following form:

$$MSD(n\tau)=2dDn\tau+2\sigma^{2}-4dDR\tau$$

where $d$ is the number of dimensions, $D$ is the diffusion coefficient, $\tau$ is the frame time, $n$ is the number of considered time step, $\sigma^{2}$is the standard deviation of the measured position for an immobile particle, $R$ is the motion blur coefficient which depends on the temporal illumination profile during the camera integration time, with $0\leq R\leq\frac{1}{4}$ and $R=\frac{1}{6}$ for the case of full-frame averaging, i.e. illumination during the entire integration time [1, 2]. In our analysis, we calculated the short-time diffusion coefficient D_12_ from the slop of the line connecting the first two points ($n$ = 1 and 2) of the MSD function (as previously reported [3–5]). The constant offsets do not influence such slope and consequently have no effects on the D_12_ estimation.

We tried therefore to try to understand theoretically the origin of the shifts of the estimated D shown in Fig. 3, starting from the simple effect of confinement. A first often used approximation for a point diffusing within a square of side $L$ [4], reported below, reproduces correctly the limits at 0 time lag $\tau$ and at very high$\tau$:

$$\mathrm{MSD}_{appr}\left( \tau\right)=\frac{L^{2}}{3}\left( 1-ⅇ^{-\frac{12D_{S}\tau}{L^{2}}} \right).$$

The short lag D (D_12_) at time resolution Δt can be calculated as $\left( \mathrm{MSD}_{appr}\left( 2\Delta t \right)-\mathrm{MSD}_{appr}\left( \Delta t \right) \right)/{4\Delta t}$, and the results for its ratio with the simulation diffusivity D_S_ is reported as thin lines in Fig. S4 (together with the data of Fig. 3 in the main text) at different Δt with colors as the one of the results of the analyses on simulations. We notice that this theoretical prediction overestimates largely the D_peak_ estimated from the simulation analyses (it underestimates the shifts), and this means that this approximation is most probably not accurate already at relatively small Δt.

In order to calculate with a better approximation the low-$\tau$ behavior of the MSD of a diffusing particle confined in a square, we calculated the probability distribution for the shifts of a point particle that moves (in 1D) according to a Gaussian distribution of width $\sigma$ but has to reflect at positions 0 and $L$; neglecting multiple reflections (a good approximation if $\sigma\ll L$), the probability distribution for a particle to arrive at position $x$ starting from position $x'$ is:

$$\frac{\theta\left( x \right)\theta\left( L-x \right)}{\sqrt{2\pi}\sigma}\left( ⅇ^{-\frac{\left( x-x^{'} \right)^{2}}{2\sigma^{2}}}+ⅇ^{-\frac{\left( x+x^{'} \right)^{2}}{2\sigma^{2}}}+ⅇ^{-\frac{\left( 2L-x-x^{'} \right)^{2}}{2\sigma^{2}}} \right),$$

where $\theta\left( x \right)$ is the Heaviside theta function with value 0 for $x$<0 and 1 for $x$>0.

We can calculate the mean square displacement averaging on the initial position $x'$, which has a uniform distribution between 0 and $L$:

$$\mathrm{MSD}_{\sigma,L}=\frac{1}{\sqrt{2\pi}\sigma L}\int_{0}^{L} \int_{0}^{L} \left( x-x^{'} \right)^{2}\left( ⅇ^{-\frac{{(x-x^{'})}^{2}}{2\sigma^{2}}}+ⅇ^{-\frac{{(x+x^{'})}^{2}}{2\sigma^{2}}}+ⅇ^{-\frac{{(2L-x-x^{'})}^{2}}{2\sigma^{2}}} \right)ⅆxⅆx^{'},$$

eventually normalized by dividing by $\frac{1}{\sqrt{2\pi}\sigma L}\iint_{0}^{L} \left( ⅇ^{-\frac{{(x-x^{'})}^{2}}{2\sigma^{2}}}+ⅇ^{-\frac{{(x+x^{'})}^{2}}{2\sigma^{2}}}+ⅇ^{-\frac{{(2L-x-x^{'})}^{2}}{2\sigma^{2}}} \right)ⅆxⅆx^{'}$ if the probability of multiple reflections is not completely negligible (for the data reported in Fig. S4, there were no differences with or without this normalization). The final $\mathrm{MSD}_{conf}$ for the motion in two dimensions is twice the $\mathrm{MSD}_{\sigma,L}$ for $\sigma=\sqrt{2D_{s}\tau}$, and the results reported in Fig. S4 are $\left( \mathrm{MSD}_{conf}\left( 2\Delta t \right)-\mathrm{MSD}_{conf}\left( \Delta t \right) \right)/\left( 4D_{s}\Delta t \right)$ for $L=16$ µm. We can notice that both the direction of the shift and its order of magnitude are correct, and in particular at the highest considered D_S_ the value of this theoretical prediction and the empirical results are almost perfectly in agreement; however, in this case the effect seems to be slightly overestimated at lower D_S_ (the resulting D_12_ is underestimated with respect to D_peak_).

We tried to explain theoretically also the results reported as filled dots in Fig. 3 of the main text, calculated starting by tracking over exact positions for a particle density of 0.3 spots/µm^2^. We considered that the diffusivity gets underestimated in such case because the tracking algorithm could prefer to link a spot in a track *A* at position $\vec{o}$ in frame *i* not to the spot in the same trajectory *A* in frame *i*+1, but to another spot in a different trajectory *B* because it is closer. If the closer spot to the position $\vec{o}$ (excluding the one in trajectory A) in the frame *i*+1 is at a distance $r_{M}$, considering a mean step in a trajectory $\sigma=\sqrt{2D_{s}\Delta t}$, and if the tracking algorithm always assigns the closest spot, then the probability distribution for the positive step length $r$ of the assigned link can be considered as a Gaussian one up to $r_{M}$, and if $r$ would be bigger than $r_{M}$ (with probability $ⅇ^{-\frac{{r_{M}}^{2}}{2\sigma^{2}}}$), the resulting $r$ should be substituted with $r_{M}$. This probability distribution can be written as:

$$P\left( r^{2}; \sigma,r_{M} \right)dr^{2}=\theta\left( {r_{M}}^{2}-r^{2} \right)\frac{1}{2\sigma^{2}}ⅇ^{-\frac{r^{2}}{2\sigma^{2}}}dr^{2}+ⅇ^{-\frac{{r_{M}}^{2}}{2\sigma^{2}}}\delta\left( {r_{M}}^{2}-r^{2} \right)dr^{2},$$

where a 2D motion has been considered by integrating over the possible angle, and $\delta\left( x \right)$ is the Dirac delta distribution.

The average $r^{2}$ in such a case is:

$$\left\langle r^{2} \right\rangle_{\sigma,r_{M}}=\int_{0}^{+\infty} r^{2}P\left( r^{2}; \sigma,r_{M} \right)dr^{2}=2\sigma^{2}\left( 1-ⅇ^{-\frac{{r_{M}}^{2}}{2\sigma^{2}}} \right).$$

This value has to be averaged over the distribution for $r_{M}$; its cumulative distribution corresponds to the probability of not having any spot at distance below $r_{M}$, i.e. in an area $\pi{r_{M}}^{2}$, for an homogeneous distribution with density $\rho$; this is the value for founding no particles in a Poissonian distribution with average number of particles $\pi{r_{M}}^{2}\rho$, i.e. $ⅇ^{-\pi{r_{M}}^{2}\rho}$. Taking the derivative with respect to $r_{M}$ to have the probability distribution, the average for the square of the assigned link length $r^{2}$ with average step in trajectory $\sigma$ ($=\sqrt{2D_{s}\Delta t}$) and density of spots $\rho$ is:

$$\left\langle r^{2} \right\rangle_{\sigma,\rho}=\int_{0}^{+\infty} \left\langle r^{2} \right\rangle_{\sigma,r_{M}}{2\pi r_{M}\rhoⅇ}^{-\pi{r_{M}}^{2}\rho}dr_{M}=\int_{0}^{+\infty} 4\pi{\sigma^{2}\left( 1-ⅇ^{-\frac{{r_{M}}^{2}}{2\sigma^{2}}} \right)r}_{M}\rhoⅇ^{-\pi{r_{M}}^{2}\rho}dr_{M}=\frac{2\sigma^{2}}{1+2{\pi\rho\sigma}^{2}}.$$

The quantities reported as dotted lines in Fig. S4 is $\left( \left\langle r^{2} \right\rangle_{\sqrt{2D_{s}\Delta t},\rho} \right)/\left( 4D_{s}\Delta t \right)$. We see that these theoretical predictions are already underestimated with respect to the D_peak_ from tracking on exact positions (the corrections to D are overestimated), and the error would be even bigger if it would have been calculated considering the differences of MSD between 2Δt and Δt. This can be explained by the fact that the above argument considers an "optimization" of the costs over a single trajectory, whereas the tracking algorithm we used tries to achieve a global optimization over all trajectories; therefore, even considering the simplest cases, the two closest points between two (consecutive) frames will not always be connected, since this could lead to a reduction in costs for one trajectory, but a potentially larger increase for another. While in the simplest case of only Brownian distribution and not too high density, one could try to consider also the optimisation of the other trajectory to calculate the probability for a track to continue with the closest spot in the following frame instead of the right one, the details would depend too much on the final implementation of the tracking algorithm.

Considering already this shortcoming, a theoretical calculation in the case of uncertainties in the spots positions and of missing spot for trying to explain the results shown in Fig. 6 would be even more difficult and more dependent on the tracking algorithm, especially if it tries to close gaps due to missed detections like the one used in u-track and therefore by us. However, there are indications that errors associated with the missing spots are indeed responsible, at least in part, for the observed broadening of the estimated diffusivity distributions (and therefore for a positive shift for its peak, see Supplementary Note 1 above). Indeed, the fractions of detected particles over the simulated ones reported in Fig. S7, if compared with the results of Fig. 6, suggest that as this fraction decreases, both the width of the D distribution and its peak position tend to increase (especially at the longest frame times).

## Supplementary Discussion

Static and dynamic errors in SPT (see also the Introduction in the main text and the first part of Supplementary Note 2) have been investigated in the literature considering their effects on localization error and analysing their impact on the Mean Square Displacement (MSD) function. The theory has been developed especially for the case of pure Brownian motion. However, as shown at the beginning of Supplementary Note 2, the results of this theory do not affect the value of the short-lag-time diffusivity as estimated by us. Our study shows therefore that this portion of theory is not sufficient to explain the effects we observed. Indeed, the temporal resolution in SPT affects static and dynamic errors in the localization phase of the spots, but a combination of additional factors, also linked to the tracking step, are relevant. These have not been investigated in depth and do not have a comprehensive explanation yet. One of these factors are the errors in the connections of spots (as shown in the section concerning the tracking on exact simulated positions in the main text), which depend on the combination of temporal resolution, particle density and diffusivity. As shown in Supplementary Note 2 above, the theoretical prediction of the influence of this factor on the estimation of the diffusivity is not easy and depends on the chosen tracking algorithm.

Other factors are connected to both the localization and tracking phase (see the section concerning the detection and tracking on simulated movies in the main text); when the SNR is too low (e.g. when the integration time is too small), the static localization error increases but also a significant portion of the spots cannot be detected; a large number of missed spots has an impact on the tracking step: if in a track the spot is detected in a certain frame, but it is not detected in the following one(s), such spot can be erroneously linked to a spot of a different track, likely more distant (depending on temporal resolution, particle density and diffusivity) and so the diffusivity can be overestimated. This kind of behaviour is confirmed by the fact that in each graph of Fig. 6 the diffusivity estimates are always larger at lower SNR than at higher SNR. Also motion blurring can cause missed detections; indeed, it alters the shape of the spots and at a certain point it causes a deviation from the Gaussian profile used by most localization algorithms (including u-track) to fit the point-spread-function. Thus, on one hand, motion blurring has a greater effect on the detection of faster-moving spots and can therefore cause a bias towards an underestimation of the diffusivity because faster tracks cannot be reconstructed correctly; on the other hand, similarly to the case of low SNR, if some of the spots are missing, connections longer than the true one can cause an overestimation of the diffusivity. To this effect is always added that caused by the logarithmic scale used for diffusivity, as explained in the supplementary notes above, which shifts the peak of the distribution towards higher diffusivity if its width is not negligible with respect to its peak value. Theoretical predictions of these effects are not easily feasible in general because of the complex interplay of different phenomena, but also because of the specific behaviour of the used tracking algorithms and software, which show different sensitivities to tracking errors, motion blurring, localization errors and uncertainties [6, 7].

## Supplementary References

1. Berglund AJ (2010) Statistics of camera-based single-particle tracking. Phys Rev E Stat Nonlin Soft Matter Phys 82:. https://doi.org/10.1103/PhysRevE.82.011917

2. Lagerholm BC, Andrade DM, Clausen MP, Eggeling C (2017) Convergence of lateral dynamic measurements in the plasma membrane of live cells from single particle tracking and STED-FCS. J Phys D Appl Phys 50:. https://doi.org/10.1088/1361-6463/AA519E

3. Marchetti L, Bonsignore F, Gobbo F, et al (2019) Fast-diffusing p75 NTR monomers support apoptosis and growth cone collapse by neurotrophin ligands. Proceedings of the National Academy of Sciences 116:21563–21572. https://doi.org/10.1073/pnas.1902790116

4. Callegari A, Luin S, Marchetti L, et al (2012) Single particle tracking of acyl carrier protein (ACP)-tagged TrkA receptors in PC12nnr5 cells. J Neurosci Methods 204:82–86. https://doi.org/10.1016/j.jneumeth.2011.10.019

5. Marchetti L, Callegari A, Luin S, et al (2013) Ligand signature in the membrane dynamics of single TrkA receptor molecules. J Cell Sci 126:4445–4456. https://doi.org/10.1242/jcs.129916

6. Hansen AS, Woringer M, Grimm JB, et al (2018) Robust model-based analysis of single-particle tracking experiments with spot-on. Elife 7:. https://doi.org/10.7554/eLife.33125

7. Chenouard N, Smal I, De Chaumont F, et al (2014) Objective comparison of particle tracking methods. Nat Methods 11:281–289. https://doi.org/10.1038/nmeth.2808
